# In Vitro Digestion of Grape Seed Oil Inhibits Phospholipid-Regulating Effects of Oxidized Lipids

**DOI:** 10.3390/biom10050708

**Published:** 2020-05-02

**Authors:** Sarah Fruehwirth, Sofie Zehentner, Mohammed Salim, Sonja Sterneder, Johanna Tiroch, Barbara Lieder, Martin Zehl, Veronika Somoza, Marc Pignitter

**Affiliations:** 1 Department of Physiological Chemistry, Faculty of Chemistry, University of Vienna, 1090 Vienna, Austria; sarah.f.fruehwirth@univie.ac.at (S.F.); sofie.zehentner@univie.ac.at (S.Z.); mohammedsalim123@hotmail.com (M.S.); sonja.sterneder@univie.ac.at (S.S.); johanna.tiroch@univie.ac.at (J.T.); barbara.lieder@univie.ac.at (B.L.); veronika.somoza@univie.ac.at (V.S.); 2 Department of Analytical Chemistry, Faculty of Chemistry, University of Vienna, 1090 Vienna, Austria; martin.zehl@univie.ac.at

**Keywords:** grape seed oil, in vitro digestion, LC-MS, lipid oxidation, phospholipids, gastric cells

## Abstract

The intake of dietary lipids is known to affect the composition of phospholipids in gastrointestinal cells, thereby influencing passive lipid absorption. However, dietary lipids rich in polyunsaturated fatty acids, such as vegetable oils, are prone to oxidation. Studies investigating the phospholipid-regulating effect of oxidized lipids are lacking. We aimed at identifying the effects of oxidized lipids from moderately (18.8 ± 0.39 meq O_2_/kg oil) and highly (28.2 ± 0.39 meq O_2_/kg oil) oxidized and in vitro digested cold-pressed grape seed oils on phospholipids in human gastric tumor cells (HGT-1). The oils were analyzed for their antioxidant constituents as well as their oxidized triacylglycerol profile by LC-MS/MS before and after a simulated digestion. The HGT-1 cells were treated with polar oil fractions containing epoxidized and hydroperoxidized triacylglycerols for up to six hours. Oxidized triacylglycerols from grape seed oil were shown to decrease during the in vitro digestion up to 40% in moderately and highly oxidized oil. The incubation of HGT-1 cells with oxidized lipids from non-digested oils induced the formation of cellular phospholipids consisting of unsaturated fatty acids, such as phosphocholines PC (18:1/22:6), PC (18:2/0:0), phosphoserine PS (42:8) and phosphoinositol PI (20:4/0:0), by about 40%–60%, whereas the incubation with the in vitro digested oils did not affect the phospholipid metabolism. Hence, the gastric conditions inhibited the phospholipid-regulating effect of oxidized triacylglycerols (oxTAGs), with potential implications in lipid absorption.

## 1. Introduction

The intake of high amounts of polyunsaturated fatty acids (PUFAs) is considered healthy. Cold-pressed grape seed oils are rich in PUFAs, especially in linoleic acid. While saturated and monounsaturated fatty acids can be biosynthesized in humans, *n*-3 and *n*-6 PUFAs should be provided by diet [1]. It is well known that the fatty acid composition of a diet can influence the fatty acid composition of membranes [2], which applies to *n*-3, *n*-6 and *n*-9 PUFAs, as their dietary intake is reflected in the cell membrane phospholipids after ingestion [2]. However, PUFAs are sensitive to lipid oxidation [1,3]. Oxidation processes influence not only the sensory [4] but also the nutritional quality of oils since oxidation products are hypothesized to provide harmful health effects as well [1,5]. The impact of dietary oxidized lipids on the composition of phospholipids in gastrointestinal cells has not previously been investigated.

Phospholipids play an important role in building up biological membranes, where they form a continuous double layer of lipid molecules to compartmentalize cells and to harbor membrane proteins which facilitate biological functions, such as transport and signaling [6]. It could be demonstrated that the passive absorption of fatty acids was facilitated when phospholipids with polyunsaturated fatty acids were increased in enterocytes and that the absorption was restricted when the membranes consisted of mainly saturated fatty acids [7].

Results indicated that linoleic acid and its primary peroxidation product, 13-hydroperoxy-9Z,11E-octadecadienoic acid (13-HpODE), are converted into the corresponding hydroxy fatty acid, 13-hydroxy-9Z,11E-octadecadienoic acid (13-HODE), which is absorbed by human gastric tumor (HGT-1) cells after a six-hour incubation period [8]. Thus, and because it could be shown that lipid absorption is initiated in the stomach, HGT-1 cells were selected as a suitable model to evaluate the influence of oxidized lipids on the phospholipid composition in the current study [9,10]. The gastrointestinal tract is constantly exposed to dietary oxidized food compounds [5]. Furthermore, it contains absorbed oxygen, is constantly at 37 °C and has a low pH [10]. The stomach and its gastric fluid were reported to be a medium for further dietary lipid oxidation. As lipid hydroperoxides are not only formed in foods, but were shown to be generated during digestion [10], it is crucial to know if the in vitro digestion of oxidized oils influences oxidized lipids and how such changes affect the composition of cellular phospholipids, as the PUFAs of phospholipids are the major target substrates of oxidation in vivo [1]. Moreover, phospholipids show a higher susceptibility to oxidation than triacylglycerols (TAGs), leading to a modulation of the cellular metabolism [1].

Grape seed oils were determined appropriate for this study, as they oxidize easily according to their fatty acid profile, which is rich in n-6 fatty acids [11], and the low amounts of antioxidants, especially polyphenols [12]. Hence, the influence of moderately versus highly oxidized grape seed oils on the phospholipid composition was analyzed.

The aim of this study was to investigate the influence of oxidized lipids from cold-pressed grape seed oils subjected to in vitro digestion on the phospholipid composition of HGT-1 cells. We hypothesized that the oxidized lipids affected the cellular phospholipid composition. Thus, the influence of the two differently oxidized grape seed oils and the in vitro digestion on the cellular phospholipid composition was evaluated.

## 2. Materials and Methods

### 2.1. Chemicals and Materials

All the chemicals were purchased from Sigma Aldrich (Vienna, Austria), Carl Roth (Karlsruhe, Germany) or VWR International GmbH (Vienna, Austria). The solvents used for the chromatography were LC-MS grade.

### 2.2. Grape Seed Oil Samples

The grape seed oils were purchased from a local producer in Retz, Austria. Two different kinds of oils were used. One oil was moderately oxidized (M_OX_) with a peroxide value of 18.8 ± 0.39 meq O_2_/kg oil, whereas the other oil was highly oxidized (H_OX_) and showed a peroxide value of 28.2 ± 0.39 meq O_2_/kg oil. The M_OX_ grape seed oil was from the authentic grape variety “Grüner Veltliner” whereas the H_OX_ grape seed oil was from the authentic grape variety “Zweigelt”. The study oils had not been treated specifically to reach these peroxide values.

The grape seeds were separated from the pomace by air separator and screening unit directly after the pressing of the juice. The seeds were dried afterwards. The oils were cold-pressed from the unfermented grape seeds using a screw extruder. Temperatures remained under 50 °C during the pressing.

The samples were stored in aliquots of 15 mL under an argon atmosphere at –20 °C until the sample preparation.

### 2.3. Study Design

**Figure 1:**
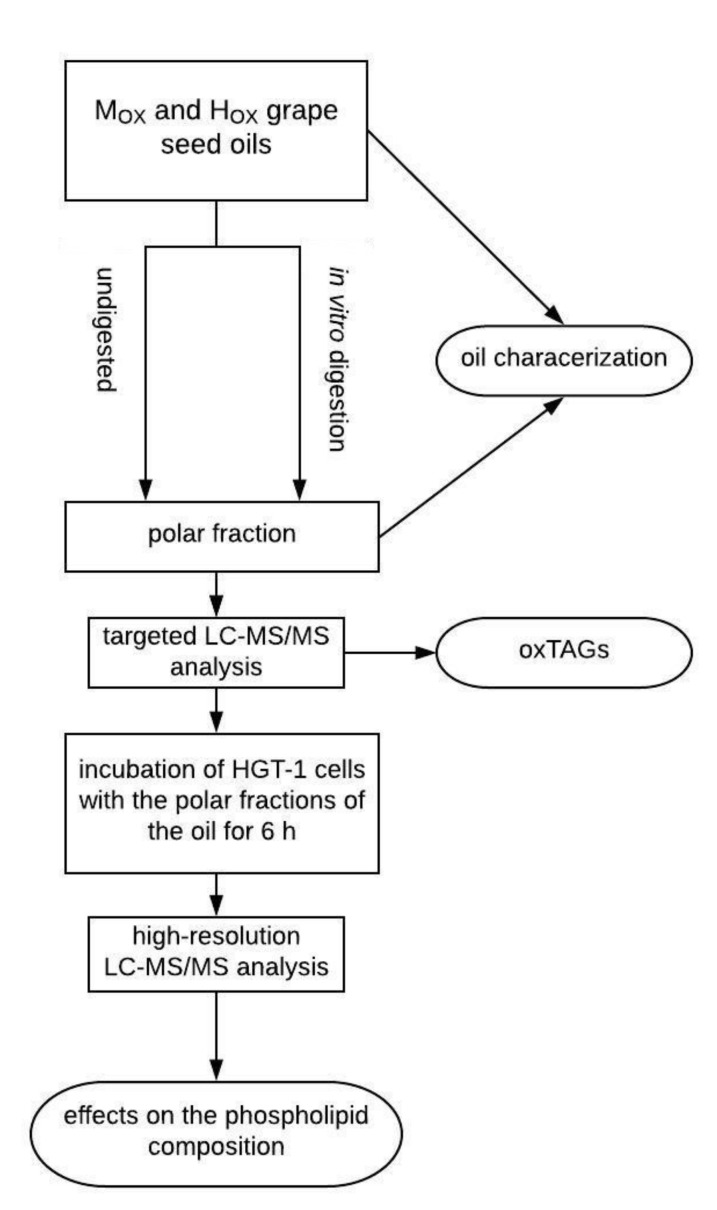
Study design.

The grape seed oil samples were digested with simulated saliva and gastric juice and incubated at 37 °C and 180 rpm for two, four and six hours (Figure 1). The polar fraction of the oils was isolated for the following analyses: (1) a targeted LC-MS/MS analysis was done to determine different oxidized triacylglycerols; (2) an incubation of the HGT-1 cells with the polar fractions of the oil for six hours followed by a high-resolution LC-MS/MS analysis, to determine the impact of digestion on the cellular phospholipid composition.

Additionally, the grape seed oil was characterized regarding the single polyphenols via high-resolution LC-MS analysis, the amount of tocopherols via HPLC/UV detection [13], the determination of fatty acids via GC/FID analysis [14] and the peroxide value using titration [15] before and after in vitro digestion.

#### 2.3.1. Gastric In Vitro Digestion Model

The gastric in vitro digestion model from Versantvoort et al. [16], which was optimized by Nieva-Echevarría et al. [17] regarding lipolysis, was used for the digestion of the grape seed oils with slight modifications. In brief, 100 mL saliva and 200 mL gastric juice were prepared freshly from the stock solutions. The saliva consisted of a mix of inorganic solutions (1 mL of 89.6 g/L KCl, 1 mL of 20 g/L KSCN, 1 mL of 88.8 g/L NaH_2_PO_4_, 1 mL of 57 g/L Na_2_SO_4_, 0.17 mL of 175.3 g/L NaCl and 2.0 mL of 84.7 g/L NaHCO_3_), an organic solution (0.8 mL of 25 g/L urea) and further components (1.5 mg uric acid and 2.5 mg mucin). For 100 mL of the gastric juice, the inorganic solutions (1.57 mL of 175.3 g/L NaCl, 0.3 mL of 88.8 g/L NaH_2_PO_4_, 0.92 mL of 89.6 g/L KCl, 1.8 mL of 22.2 g/L CaCl_2_ × 2 H_2_O, 1.0 mL of 30.6 g/L NH_4_Cl and 0.65 mL HCl 37%), the organic solution (0.34 mL of 25 g/L urea) and further components (0.25 g pepsin from porcine gastric mucosa, 83 mg Amano Lipase A from *Aspergillus niger*, 0.3 g mucin from porcine stomach type 2) were mixed together.

To produce 100 mL of each solution, the inorganic solutions and the urea solution were pipetted into 50 mL tubes and brought to a final volume of 50 mL with bidistilled water. Both solutions were mixed in a beaker and the further components were added. Then, the pH value (6.80 ± 0.20 for the saliva and 1.30 ± 0.02 for the gastric juice) was adjusted using 37% hydrochloric acid.

The gastric in vitro digestion was performed for two, four and six hours as lipids were detected in the stomach until six hours after ingestion [18]. For each oil variety, 4.5 g of oil were pipetted into 50 mL tubes. A total of 6 mL simulated saliva was added to the oil, vortexed and incubated at 37 °C for five minutes. Subsequently, 12 mL simulated gastric juice was added. The mixture was vortexed for ten seconds and incubated at 37 °C and at 180 rpm without light for two, four or six hours. After digestion, the samples were centrifuged for ten minutes at 4500× *g* at 4 °C (Centrifuge 5804 R, Eppendorf, Vienna, Austria). The oily phase was stored in aliquots under the argon atmosphere at −80 °C until sample preparation.

#### 2.3.2. Sample Preparation

Amber glass vials were cleaned according to Grüneis et al. [14] to remove all traces of lipids and lipid-oxidation-promoting agents.

Sep-Pak silica columns (Strata SI-1 Silica, 500 mg, Phenomenex, Aschaffenburg, Germany) were used to isolate the polar fraction of the grape seed oil. This was performed according to the protocol described by Márquez-Ruis et al. [19] with some modifications. First, the cartridges were conditioned before use by rinsing with 10 mL petroleum ether/diethyl ether (90/10). Secondly, 50 µL of the oil was dissolved in 1 mL petroleum ether/diethyl ether (90/10) and loaded on the column. The non-polar fraction was eluted with 2 mL petroleum ether/diethyl ether (90/10) whereas the second fraction, which contained the polar compounds, was eluted with 2 mL diethyl ether. The polar fraction was evaporated with nitrogen and dissolved in 0.5 mL 2-propanol.

Samples were stored at –20 °C under argon atmosphere until analysis.

### 2.4. Characterisation of the Grape Seed Oils

#### 2.4.1. GC/FID Analysis of the Fatty Acid Composition

The fatty acid composition of the grape seed oils was analyzed before and after the gastric in vitro digestion by means of GC/FID (GC-2010 Plus, Shimadzu, Korneuburg, Austria). The fatty acids were detected as their respective fatty acid methyl esters (FAME). The sample preparation and GC/FID analysis were done according to Grüneis et al. [14]. In brief, a total of 100 mg oil was pipetted into a 50 mL tube, spiked with 0.5 mL heptadecanoic-acid-methylester-solution (1%) as the internal standard, before adding 2 mL toluol, 100 mg pyrogallol and 4 mL sodium methoxide (0.5 M). After vortexing under an argon atmosphere at 50 °C for 12 min, the samples were cooled in the fridge for five minutes. A total of 200 µL acetic acid (99%), 5 mL bidistilled water and 5 mL n-hexane were added. The phases were separated after vortexing for two minutes and the n-hexane phase was collected. The aqueous phase was extracted with 5 mL n-hexane again and the n-hexane phase was collected. A total of 9 mL (2 × 4.5 mL) of hexane was collected and considered in the calculation. Before the analysis, sodium sulphate was used to dry the n-hexane phase and the samples were passed through the 0.45 µm polyvinylidene fluoride (PVDF) filters. The following temperature gradient was used: 60 °C for 2 min, increased by 13 °C/min until 150 °C and by 2 °C/min until 240 °C.

For quantitation, the calibration curves were measured for palmitic acid, stearic acid, oleic acid, linoleic acid and α-linolenic acid, which were the most abundant fatty acids in the grape seed oils. The limit of detection (LOD) and the limit of quantitation (LOQ) were determined using the blank determination method [20,21]. The LOD was 0.2 µg/mL and the LOQ was 0.5 µg/mL.

The recovery rate was evaluated using a 1% heptadecanoic-acid-methylester internal standard, which was added to every sample. The average recovery rate for the oil samples which were not digested was 90.94% ± 6.43% whereas the recovery rate for the in vitro digested oil samples was 99.50% ± 3.65%.

#### 2.4.2. Peroxide Value

The peroxide value was measured before and after the gastric digestion of the oils according to the method of Wheeler [15]. In brief, 5.0 g of oil were dissolved in acetic acid/chloroform (3:2, v/v). Then, 0.5 mL of a saturated potassium iodide solution were added and the sample was shaken for one minute. Afterwards, 30 mL bidistilled water and starch were added. The samples were titrated using a 0.1 N sodium thiosulfate solution until complete discoloration was reached. The blank test was performed without oil.

#### 2.4.3. HS-GC/MS Quantitation of Hexanal

The content of hexanal in the M_OX_ and H_OX_ grape seed oils was quantified according to Giuffrida et al. [22] with slight modifications according to Pignitter et al. [23]. The extraction of hexanal was performed with 20 mL acetone and water (70:30, *v*/*v*). The samples were vortexed for 2 min, homogenized at room temperature for 1 h in a rotator and subsequently centrifuged at 4 °C and 2250× *g* for 2 min. The supernatant was collected for analysis and the extraction was repeated three times. D12-hexanal (4 µg/mL) was added as the internal standard to the first extraction step.

The LOD (S/N = 3) and the LOQ (S/N = 10) were determined using the signal-to-noise-ratio [20]. The LOD was 0.02 µg/mL and the LOQ was 0.04 µg/mL.

#### 2.4.4. Extraction and High-Resolution LC-MS Analysis of Polyphenols

The extraction of polyphenols was done according to Singleton and Rossi [24] with some modifications. A total of 2.5 g grape seed oil was dissolved in 2.5 mL hexane. To extract the phenols, 1 mL methanol/H_2_O (80:20, *v*/*v*) was added and vortexed for four minutes. The samples were centrifuged for five minutes at 2880× *g* and 4 °C. The aqueous phase was collected. Extraction and centrifugation were done thrice until a final volume of 3 mL was collected. The extracted samples were washed with hexane.

The methanol phases were evaporated with N_2_ and the aqueous phases were freeze-dried. The samples were resolved in 100 µL methanol/H_2_O (80:20, *v*/*v*).

The extracted polyphenolic samples were injected (5 µL) into a Vanquish UHPLC system (Thermo Fisher Scientific, Germering, Germany) and separated on a C18 column (Acclaim 120, 2.1 mm × 150 mm, 3 µm, Thermo Fisher Scientific, Germering, Germany) at 25 °C.

The mobile phase was acetonitrile with 0.1% formic acid (A) and H_2_O (LC-MS grade) with 0.1% formic acid (B). The HPLC gradient elution was programmed as follows: 0–2 min with an isocratic flow at 10% B, 2–8 min from 10% to 80% B, 8–20 min to 100% B, holding for 1 min, 21–21.1 min from 100% to 10% B and an isocratic flow of 10% B from 21.1–25 min. The flow rate was set to 0.3 mL/min.

The HPLC was coupled to a Dual-Pressure Linear Trap-Quadrupole-Orbitrap mass spectrometer (Thermo Fisher Scientific, Germering, Germany). The following electrospray ionization (ESI) ion source settings were applied: source voltage: 3.5 kV, capillary voltage: 25 V, capillary temperature: 300 °C, sheath gas flow: 45 AU (N_2_), aux gas flow: 10 AU (N_2_).

Each sample was analyzed in negative mode in the range of *m*/*z* 100–1000. Calibration was performed using *m*/*z* 112.9856, ((HCOOH)_2_+Na-2H)^-^ as lock mass.

#### 2.4.5. HPLC/UV Analysis of Tocopherols

The tocopherols were analyzed as described in a previous work [13], with some modifications as follows. The grape seed oil (50 mg) was dissolved in 1 mL 2-propanol and 1 µL tocol (5 µg/mL) was added as the internal standard. The samples were filtered through a 0.2 µm nylon-filter and analyzed twice. The samples (20 µL) were injected into an HPLC system (Ultimate 3000 RS, Dionex/Thermo Fisher Scientific, Germering, Germany) coupled to a diode array detector (DAD 3000 RS, Dionex/Thermo Fisher Scientific, Germering, Germany) and separated on a C18 column (Kinetex 5 µm, EVO C18, 150 mm × 4.6 mm, Phenomenex, Aschaffenburg, Germany) at 10 °C with a flow rate of 0.5 mL/min. The mobile phase was bidistilled water (A) and methanol (B).

The HPLC gradient elution was programmed as follows: 0–4 min from 95% to 100% B, holding for 10 min, 14–16 min from 100% to 95% B and an isocratic flow of 95% B from 16–18 min.

For quantitation, the calibration curves were measured in concentrations from 5–500 µg/mL for α-tocopherol (R^2^ = 0.9994, LOD = 1.25 µg/mL, LOQ = 4.18 µg/mL), γ-tocopherol (R^2^ = 0.9998, LOD = 0.41 µg/mL, LOQ = 1.37 µg/mL) and δ-tocopherol (R^2^ = 0.9999, LOD = 1.13 µg/mL, LOQ = 3.37 µg/mL). The LOD (S/N = 3) and the LOQ (S/N = 10) were determined using the signal-to-noise-ratio [20].

The recovery rate was calculated using tocol as internal standard to be 119.78% ± 11.73%.

### 2.5. Cell Culture

Human gastric tumor cells (HGT-1) were obtained from C. Laboisse (Laboratory of Pathological Anatomy, Nantes, France). HGT-1 cells are a well established cell model for investigating the effects of specific substances on the mechanism of gastric digestion [25,26]. The cells were cultivated in Dulbecco’s Modified Eagle’s Medium (DMEM) supplemented with 10% (*v*/*v*) fetal bovine serum (FBS) (Gibco, Thermo Fisher Scientific) and 1% (*v*/*v*) penicillin/streptomycin (Sigma) under the standard conditions (37 °C, 5% CO_2_ in a humidified incubator) as described before [25,26] and then seeded 20 h before the measurement with a density of 3 × 10^6^ cells per well in a transparent six well plate (Sarstedt, Nümbrecht, Germany).

#### 2.5.1. Cell Viability

The cell viability was evaluated using the 3-(4,5-dimethylthiazol-2-yl)-2,5-diphenyltetrazolium bromide (MTT) assay as described in a previous work [27]. The HGT-1 cells were treated with the polar fraction of the oil samples for six hours. The absorbance was measured at 570 nm and at the reference wavelength of 650 nm using the Infinite 200 Pro Plate Reader (Infinite M200, Tecan, Männedorf, Switzerland). The cell viability was calculated relative to the control cells which were treated with solvent control only (medium plus 0.1% isopropanol, untreated cells = 100%).

#### 2.5.2. Incubation of HGT-1 Cells and Cellular Extraction of Phospholipids

The HGT-1 cells were starved for one hour with DMEM (2% (*w*/*v*) bovine serum albumin (BSA) (Carl Roth)) at standard conditions synchronizing the basal activity [28]. Afterwards, the cells were incubated for six hours with the polar fraction of the grape seed oil (1:5000) diluted in 2-propanol (0.1% final concentration on the cells). The extraction of phospholipids was done according to Zhang et al. [29]. After washing with phosphate-buffered saline (PBS), the cells were lysed with 300 µL H_2_O and subjected to three freeze-thaw cycles. The lysate was centrifuged at 16,000 × *g* and 4 °C for 5 min and a total of 5 µL was taken for the Bradford assay [19] to determine the protein content of the HGT-1 cell lysate. The protein content was used to normalize the peak areas. The results were depicted as treated over control (%). For the precipitation of proteins and the extraction of phospholipids from the cell lysate, 250 µL ice-cold methanol was added before centrifugation at 16,000 × g and 4 °C for 5 min. The supernatant was evaporated with nitrogen and the aqueous phase was freeze-dried.

The samples were stored at −80 °C under argon atmosphere until the analysis with high-resolution LC-MS/MS.

### 2.6. LC-MS and LC-MS/MS Analysis

#### 2.6.1. Targeted Analysis by LC-MS/MS

Targeted LC-MS/MS analysis was done according to Grüneis et al. [14]. The samples, which consisted of the polar fraction of the grape seed oil, were diluted 1:400 with 2-propanol and injected (2 µL) into a LC-MS system (LCMS-8040, Shimadzu, Korneuburg, Austria). They were separated on a C18 column (Kinetex EVO, 150 × 3.0 mm, 5 µm, Phenomenex, Aschaffenburg, Germany). The mobile phase consisted of acetonitrile/H_2_O (60/40) with 0.1% formic acid and 10 mM ammonium formate (A) and acetonitrile/isopropanol (20/80) with 0.1% formic acid and 10 mM ammonium formate (B).

The following HPLC gradient was used: 0–8 min with 60% B to 100% B, 8–28 min 100% B, 28–30 min 100% to 60% B and 30–35 min 60% B. The flow rate was set to 0.5 mL/min.

The HPLC was coupled to a triple quadrupole MS with an ESI source. The MS instrument was operated in multiple reaction mode (MRM). The subsequent MS settings were used: nebulizing gas flow 3 L/min, drying gas flow 12 L/min, desolvation line temperature 250 °C and heat block temperature 350 °C. Argon was used as the collision-induced dissociation (CID) gas with a collision energy of 20 eV. To cope with the carry-over effects, 2-propanol samples were measured as blanks before every sample and a washing step with dichlormethane as a solvent was performed at 0.5 mL/min for 20 min after every sample.

Oxidized triacylglycerols (oxTAGs) in grape seed oil were analyzed via MS fragmentation pathways [14], the METLIN database and the MoNa–MassBank of North America. The corresponding MRM transitions are listed as the (M + NH_4_)^+^ adducts in Table S1.

For quantitation, the calibration curves were performed according to Grüneis et al. [14] using a glyceryltriheptadecanoate standard. The LOD and the LOQ were determined using the blank determination method. [20,21]. The LOD was 0.1 nM and the LOQ was 0.2 nM for hydroperoxidized as well as epoxidized TAGs.

To determine the free linoleic acid, linoleic acid hydroxide and linoleic acid hydroperoxide, an analysis was performed in negative ion mode. The MRM transitions were measured using the (M − H)^−^ ions (Table S2) [30].

#### 2.6.2. High-Resolution LC-MS and LC-MS/MS Analysis

The extracted samples of HGT-1 cells, which were treated and incubated with the polar fractions of the oils, were injected (2 µL) into a Vanquish UHPLC system (Thermo Fisher Scientific, Germering, Germany) and separated on a C18 column (Acclaim 120, 2.1 mm × 150 mm, 3 µm, Thermo Fisher Scientific, Germering, Germany) at 25 °C.

The mobile phase was acetonitrile/H_2_O (50/50) with 0.1% formic acid and 5 mM ammonium formate (A) and acetonitrile/isopropanol (5/95) with 0.1% formic acid and 5 mM ammonium formate (B).

The HPLC gradient elution was programmed as follows: 0–2 min with an isocratic flow at 10% B, 2–8 min from 10% to 80% B, 8–20 min to 100% B, holding for 1 min, 21–21.1 min from 100% to 10% B and an isocratic flow of 10% B from 21.1–25 min. The flow rate was set to 0.2 mL/min.

The HPLC was coupled to a Dual-Pressure Linear Trap-Quadrupole-Orbitrap mass spectrometer (Thermo Fisher Scientific, Germering, Germany). The following ESI ion source settings were applied: source voltage: 3.5 kV, capillary voltage: 25 V, capillary temperature: 300 °C, sheath gas flow: 45 AU (N_2_) and aux gas flow: 10 AU (N_2_).

Each sample was analyzed in positive and negative mode in the range of *m/z* 100–1000. Calibration was performed using two different lock masses (*m/z* 226.9515, ((HCOONa)_3_ + Na)^+^, for the positive mode and *m/z* 112.9856, ((HCOOH)_2_+Na-2H)^−^, for the negative mode). Furthermore, the LC-MS experiments were performed in manual MS/MS (“MRM”) mode. The normalized collision energy (CID) was set to 35 eV for the positive and 30 eV for the negative mode.

The quality control (QC) samples consisted of 2 µL of each digested and undigested sample and were analyzed during the measurement after every fifth sample. The QC samples remained stable during the whole measurement.

To verify that no phytosterols were present in the polar fractions of the grape seed oils, the following (M + H)^+^ ions of the most common phytosterols in oil and their esters with linoleic acid were extracted from the MS^1^ data. These phytosterols and their esters with linoleic acid were also determined as (M − H)^−^ ions (Table S3).

Furthermore, the following phospholipids in the polar fraction were searched for in the MS^1^ data according to Ovcharova et al. [31] as (M + H)^+^ as well as (M − H)^−^ ions: phosphoinositols PI (18:2/18:1), PI (18:2/18:2), PI (16:0/18:1), PI (16:0/16:0), PI (16:0/18:2), phosphocholines PC (18:1/18:1), PC (14:0/14:0), and PC (14:0/18:1) (Table S3).

#### 2.6.3. Comparative Analysis of Digested and Undigested Samples

The digested (two, four or six hours, *n* = 3 for each time point) and undigested (*n* = 3) samples of each oil were compared with XCMS online. XCMS online identifies features whose relative intensity varies between sample groups and calculates p-values and fold changes [32]. MSConvert [33] was used to convert the raw data files to the mzXML format, which is an accepted file formate for XCMS online. The acquisition parameters were set as follows: fold change > 1.5, intensity > 5000, *p*-value < 0.05 and retention time deviation ≤ 5 s. The ordered bijective interpolated warping (OBI-Warp) method [34] was chosen for retention time correction whereas centWave algorithms [35] were selected for peak picking and grouping. The possible adducts ((M + H)^+^, (M + NH_4_)^+^, (M + Na)^+^ and (M + K)^+^) and their isotopic features were both considered during the analysis. A *m/z* deviation of 5 ppm was allowed for consecutive runs and a maximum peak width of 60 s was defined. Unknown features, which changed during the in vitro digestion, were further subjected to MS/MS measurements.

### 2.7. Statistical Analysis

The data were analyzed using Excel and SigmaPlot 14.0 (Synstat Software GmbH, Erkrath, Germany) and are shown as the mean ± SD. The results of the grape seed oil characterization regarding the amount of fatty acids, peroxide value and tocopherol content between the two different grape seed oils as well as the influence of the in vitro digestion on the amount of fatty acids were determined by *t*-test, or if the data did not exhibit normal distribution, via the Mann–Whitney U-Test. The effect of in vitro digestion on the peroxide value, tocopherol content and single polyphenols was evaluated via one-way analysis of variance (ANOVA) and a Holm–Sidak post-hoc test as four different time points were compared. The results from the targeted LC-MS/MS experiments were also analyzed by one-way ANOVA and Holm–Sidak post-hoc test. For the data of the high-resolution LC-MS/MS analysis, which did not show normal distribution, the ANOVA on ranks and the Student–Newmann–Keuls post-hoc test was applied. To identify the unknown features in the digested and undigested samples with an XCMS online, the unpaired parametric *t*-test (Welch *t*-test) was used.

## 3. Results and Discussion

### 3.1. Characterisation of Cold-Pressed Grape Seed Oil

The impact of the in vitro digestion on the different oxidation products in cold-pressed grape seed oils and their possible effects on phospholipids in HGT-1 cells were determined. Moreover, the study oils were characterized regarding their fatty acid compositions and peroxide values as well as their contents of antioxidants, such as tocopherols (Table 1) and single polyphenols (Table 2).

Grape seed oil is generally rich in unsaturated fatty acids, predominantly linoleic acid, which was shown to be the main fatty acid with 78.1 ± 3.86 g/100 g in the M_OX_ oil and with 74.0 ± 1.92 g/100 g in the H_OX_ oil, confirming that polyunsaturated fatty acids make more up than three-quarters of the amount of total fatty acids in grape seed oil [3,36,37]. Thus, grape seed oil is susceptible to lipid oxidation [38]. This could be confirmed by the peroxide value, which distinguished the oils as M_OX_ oil with a peroxide value of 18.8 ± 0.39 meq O_2_/kg oil and as H_OX_ oil with a peroxide value of 28.2 ± 0.39 meq O_2_/kg oil. It has to be noted that according to Codex Alimentarius [39] the limit of the peroxide value for cold-pressed oils is set to 10 meq O_2_/kg oil. However, the aim was to determine the influence of the oxidized lipids and therefore oils exceeding this threshold were used.

Additionally, hexanal was determined as the secondary lipid oxidation marker. The amount of hexanal was 13.6 ± 0.61 and 10.7 ± 0.54 µg/mL oil in the M_OX_ and H_OX_ oils, respectively. The M_OX_ oil showed a higher content of hexanal than the H_OX_ oil, even though the H_OX_ oil exhibited a higher peroxide value. This might be explained by hexanal being the dominant oxidation product of linoleic acid [40], which was higher in the M_OX_ oil than in the H_OX_ oil.

The main tocopherol in the grape seed oils was α-tocopherol with an amount of 325 ± 43.2 mg/kg oil and 329 ± 28.5 mg/kg oil in the M_OX_ oil and the H_OX_ oils, respectively. The amounts of α-tocopherol showed no difference between the M_OX_ oil and the H_OX_ oils. An amount of 52.2 ± 3.46 mg/kg oil γ-tocopherol and 80.4 ± 2.12 mg/kg oil γ-tocopherol in the M_OX_ oil and the H_OX_ oil, respectively, could be detected, whereas δ-tocopherol was below the limit of detection. α-Tocopherol is known to be the main tocopherol homologue in grape seed oil, followed by γ-tocopherol [12,36,37,41].

Although it was shown in literature [12,36,37,41] that tocopherol contents vary a lot according to different growing and processing conditions, the analyzed grape seed oils in this study showed higher contents of α-tocopherol and γ-tocopherol than the grape seed oils reported in the literature. Demirtas et al. [12] analyzed seven cold-pressed grape seed oils and showed that the α-tocopherol content varied between 98.3 and 225 mg/kg oil and the γ-tocopherol content varied between 21.5 and 33.7 mg/kg oil, whereas δ-tocopherol could not be detected. Assumpção et al. [42] detected α-tocopherol concentrations between 17.4 and 20.3 mg/kg oil and γ-tocopherol concentrations between 2.50 and 11.9 mg/kg oil. However, it could be shown that high variations of the tocopherol content occur not only during the cold pressing of the oil but as well during chemical oil extraction [36,37], where the α-tocopherol content varied between 85.5 and 244 mg/kg oil and the γ-tocopherol content between 2.50 and 45 mg/kg oil in ten Portuguese grape seed oils [41].

### 3.2. Effects of Gastric In Vitro Digestion on Cold-Pressed Grape Seed Oil

Gastrointestinal conditions have been reported to oxidize lipids and the acidic pH of the stomach to accelerate the generation of lipid hydroperoxides and their decomposition products [10,43]. Thus, the human gastric fluid may be an excellent medium for enhancing the oxidation of lipids [10], as lipid oxidation products could be formed during the preparation and digestion of food [43]. To determine the influence of the in vitro digestion on the oxidative status of the cold-pressed grape seed oil, the main antioxidants as well as the peroxides and fatty acid compositions were analyzed and compared (Table 2 and Table 3).

Interestingly, the amounts of α-tocopherol and γ-tocopherol did not change during the in vitro digestion, indicating that these tocopherols were not affected by the gastric digestion but remained stable. This is contrary to Kenmogne-Domguia et al. [44], who demonstrated that tocopherol levels were reduced in an emulsion of fish oil and sunflower oil within the first hour of digestion as they added metmyoglobin during the in vitro digestion, which acted as prooxidant in the gastric milieu. Surprisingly, the values for δ-tocopherol changed significantly (*p* < 0.05) in the H_OX_ oil during the in vitro digestion in the current study. Whereas δ-tocopherol was not quantifiable until two hours, totals of 93.6 ± 9.18 mg/kg oil and 88.6 ± 12.9 mg/kg oil were measured after four hours and six hours of the simulated digestion, respectively. It might be conceivable that δ-tocopherol was released from the matrix where it might be present in its esterified form. During digestion, the ester linkage could be cleaved, leading to an increase of free δ-tocopherol. [45,46] This increment of δ-tocopherol is most likely the reason why the total amount of tocopherols in the H_OX_ oil changed significantly by 13.26% (*p* < 0.05) from 454 ± 18.8 mg/kg oil before to 514 ± 10.9 mg/kg oil after six hours of in vitro digestion even though α-tocopherol and γ-tocopherol remained stable. Chew et al. [45] and Cheong et al. [46] were able to show similar results by analyzing kenaf seed oil, for which the authors analyzed an increase of total tocopherols. Chew et al. [45] analyzed an increase by 32.1%, whereas Cheong et al. [46] analyzed an increase by 230% after a gastrointestinal in vitro digestion for three hours. The authors explained the increase of the total amount of tocopherols with an improved bioavailability due to the migration of tocopherols from the matrix but did not report at which constituents the tocopherols were bound in the oil.

Additionally, single polyphenols were measured by high-resolution LC-MS (Table 2). Catechin, ferulic acid and p-coumaric acid were depicted as they are common polyphenols in grape seed oil [47,48]. It could be shown that these three polyphenols decreased mainly between zero and two hours of the in vitro digestion. The catechin decreased on average by 76.2% and 83.7% in the M_OX_ and H_OX_ oils, respectively. The ferulic acid decreased on average by 67.9% and 64.6%, whereas the p-coumaric acid decreased on average by 71.0% and 70.9% in the M_OX_ and H_OX_ oils, respectively. Other polyphenols, such as naringenin, kaempferol and ursolic acid, were not affected by the in vitro digestion.

The in vitro digestion-induced decline of polyphenols can be explained by (I) the free radical scavenging activity of polyphenols [49] and (II) their ability to regenerate oxidized vitamin E [50], which would explain why the main tocopherols remained stable whereas phenols decreased during digestion. Furthermore, according to Réblová and Okrouhlá [51], phenolic acids such as gallic acid, gentisic acid or protocatechuic acid, were able to protect α-tocopherol from oxidative degradation.

Apart from the antioxidants, the fatty acid composition was also shown to be affected by the in vitro digestion (Table 3). The total amount of fatty acids decreased from 99.6 ± 4.91 g/100 g oil to 93.9 ± 1.07 g/100 g oil (5.66%) in the M_OX_ oil and from 99.8 ± 2.63 g/100 g oil to 93.3 ± 0.75 g/100 g oil (6.49%) in the H_OX_ oil. These declines arose mainly from the reduction of the content of linoleic acid, which decreased (*p* < 0.05) by 5.20 g/100 g oil and 5.42 g/100 g in the M_OX_ oil and the H_OX_ oil, respectively. Therefore, the reduction of the content of polyunsaturated linoleic acid might indicate the increased oxidation processes after the in vitro digestion for six hours. Palmitic acid and stearic acid, both saturated fatty acids, remained stable during the in vitro digestion. Oleic acid, a monounsaturated fatty acid, remained stable as well in the M_OX_ oil, but not in the H_OX_ oil where it decreased during the in vitro digestion (*p* < 0.05). Even though linoleic acid changed significantly during the in vitro digestion, linolenic acid, which exhibited even one double bond more, was not affected in the M_OX_ oil. This can be explained by the extremely low content of linolenic acid in the M_OX_ oil compared to the H_OX_ oil, which was 0.36 ± 0.02 mg/100 g oil and 0.37 ± 0.01 mg/100 g oil before and after the in vitro digestion, respectively. Kenmogne-Domguia et al. [44] were able to demonstrate similar results during a simulated in vitro digestion of an emulsion of fish oil and sunflower oil for three hours. The authors showed that the amount of PUFAs changed significantly due to a decline of the n-3 fatty acids, eicosapentaenoic acid (20:5) and docosahexaenoic acid (22:6), whereas the amounts of SFAs and MUFAs remained stable [44].

**Figure 2:**
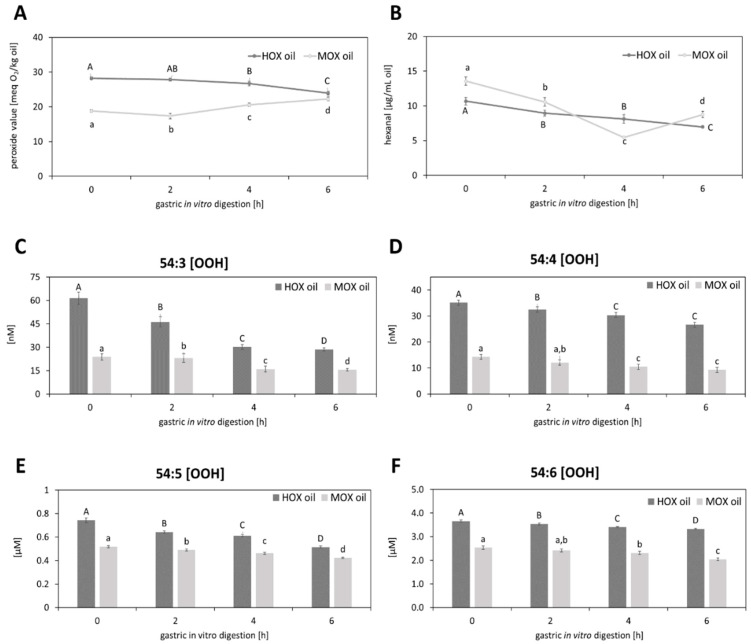
Effects of the gastric in vitro digestion on (**A**) the peroxide value (meq O_2_/kg oil), (**B**) the amount of hexanal (µg/mL) and on the hydroperoxidized triacylglycerols (OOH) (**C**) 54:3 (OOH), (**D**) 54:4 (OOH), (**E**) 54:5 (OOH), and (**F**) 54:6 (OOH) in the moderately and the highly oxidized grape seed oils. Statistically significant differences between the time of digestion (0, 2, 4, 6 h) for each oil are indicated with different letters (*n* = 3, One-Way-ANOVA, Holm–Sidak, *p* < 0.05). Capital letters indicate the significant differences between the time of digestion of the H_OX_ oil and lowercase letters mark significant differences between the time of digestion of the M_OX_ oil.

The peroxide value of the M_OX_ grape seed oil also changed (*p* < 0.05) upon digestion (Figure 2A), showing a decrease from 18.8 ± 0.39 meq O_2_/kg oil to 17.3 ± 0.83 meq O_2_/kg oil within the first two hours. Longer in vitro digestion for four and six hours led to a further increase of the peroxide value to 20.5 ± 0.49 meq O_2_/kg oil and 22.3 ± 0.60 meq O_2_/kg oil, respectively. It could be shown that the peroxide value increased after six hours of in vitro digestion but decreased after two hours. The decreased amounts of hydroperoxides might reflect the stages of progressive lipid oxidation leading to the decomposition of peroxides [52], as evident in the current study after two hours of the digestion of the M_OX_ grape seed oil. However, the exposure of the M_OX_ study oil to the simulated digestion conditions for more than two hours might have caused the lipids to become hydrolyzed, thereby releasing their respective fatty acids, which could be oxidized faster than the esterified fatty acids [4], resulting in a further increase of the peroxide value. The peroxide value considers oxidized free fatty acids and other oxidized lipids [53]. In humans, the enzymatic lipid digestion is initiated by the gastric lipase, which works optimally in a broad pH-range between 3.0 and 6.0 [54]. Consequently, about 10% to 25% of all triacylglycerols in the stomach are hydrolyzed to free fatty acids and sn-1,2-diacylglycerols [54]. Even up to 40% of triacylglycerols could be reported to be hydrolyzed to release free fatty acids [55], which have a higher susceptibility to oxidation than the triacylglycerols, which might explain the in vitro digestion-induced increase of the peroxide value in the M_OX_ oil.

Regarding the peroxide values for the H_OX_ oil, it could be determined that the peroxide value remained stable until two hours of digestion and then decreased (*p* < 0.05) after four and six hours of digestion.

The results of the peroxide value in the M_OX_ and H_OX_ oils could also be confirmed by the amounts of hexanal measured during the in vitro digestion (Figure 2B). The hexanal content in the M_OX_ oil decreased after two hours and four hours of digestion and increased after 6h of digestion (*p* < 0.05). The fact that the peroxide value in the M_OX_ oil increased after four hours but the hexanal content still decreased after four hours can be explained by the hexanal being a secondary lipid oxidation marker and being generated from the degeneration of lipid hydroperoxides [56].

The hexanal content in the H_OX_ oil decreased after two hours and four hours of digestion and decreased further after six hours of digestion, like the peroxide value.

After studying the impact of the in vitro digestion on the oxidative status of the grape seed oil, the effects on the isolated oxidized triacylglycerols in the polar fraction of the oil were analyzed. The oxidized triacylglycerol-containing fraction was used to treat the HGT-1 cells. The polar fraction did not contain tocopherols and polyphenols, nor phospholipids or phytosterols (Table S3). According to Garavaglia et al. [47] and Ohnishi et al. [11] the main phytosterols in grape seed oils are sitosterol, campesterol, stigmasterol, cholesterol and Δ5-avenasterol. These components as well as their esters with linoleic acid could not be detected by high-resolution MS. Furthermore, the polar fraction was also analyzed for linoleic acid hydroperoxide, linoleic acid hydroxide as well as free linoleic acid (Table S2). However, none of these components were detected.

**Figure 3:**
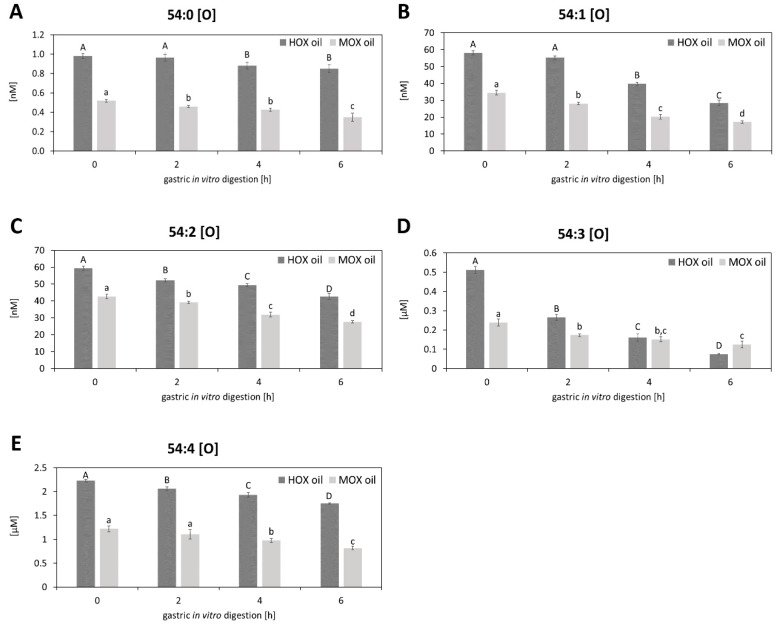
Effects of the gastric in vitro digestion on the epoxidized triacylglycerols (O) in the moderately and the highly oxidized grape seed oils: (**A**) 54:0 (O), (**B**) 54:1 (O), (**C**) 54:2 (O), (**D**) 54:3 (O) and (**E**) 54:4 (O). Statistically significant differences between the time of digestion (0, 2, 4, 6 h) for each oil are indicated by different letters (*n* = 3, One-Way-ANOVA, Holm–Sidak, *p* < 0.05). Capital letters indicate significant differences between the time of digestion of the H_OX_ oil, lowercase letters mark significant differences between the time of digestion of the M_OX_ oil.

It could be shown that oxidized lipids could be isolated in the polar fraction of the oil [14]. To evaluate the effects of the in vitro digestion on oxidized TAGs, a targeted LC-MS/MS analysis was done according to Grüneis et al. [14], where nine differently oxidized triacylglycerols (oxTAGs) could be determined in the polar fraction of the grape seed oils. A total of four oxTAGs— 54:3 (OOH), 54:4 (OOH), 54:5 (OOH) and 54:6 (OOH)—could be assigned to the oxidation class of hydroperoxides (Figure 2C–F) whereas five oxTAGs—54:0 (O), 54:1 (O), 54:2 (O), 54:3 (O) and 54:4 (O)—were identified as epoxides (Figure 3). It could be shown that the oxTAGs—hydroperoxides as well as epoxides—decreased with the advanced digestion. Hydroperoxidized TAGs decreased about 26.2% in the M_OX_ oil and about 31.7% in the H_OX_ oil after six hours of digestion whereas epoxides decreased about 39.7% in the M_OX_ oil and about 39.9% in the H_OX_ oil after the same time. Hence, gastric conditions promoted the decomposition of oxTAGs. This could be explained by the acid environment which characterizes gastric digestion. Furthermore, it could be shown that the amounts of oxTAGs increased with the increasing unsaturation (Figure 2 and Figure 3). Thus, higher amounts of PUFAs resulted in higher amounts of oxTAGs.

### 3.3. Effects of Oxidized Lipids on Phospholipid Composition in HGT-1 Cells

To evaluate whether oxTAGs or their decomposition products affect the phospholipid composition, HGT-1 cells were treated with the polar fractions of the undigested or digested study oils. The fractions had no impact on the cell’s viability by means of the cell viability assay (*p* > 0.05). As the dietary lipids were shown to be incorporated into the cellular membranes [2], the effect of the oxidized lipids on the phospholipids was investigated by high-resolution LC-MS/MS. As mentioned before, the presence of tocopherols, polyphenols, phospholipids or phytosterols above the LOD in the polar fraction could be excluded.

**Figure 4:**
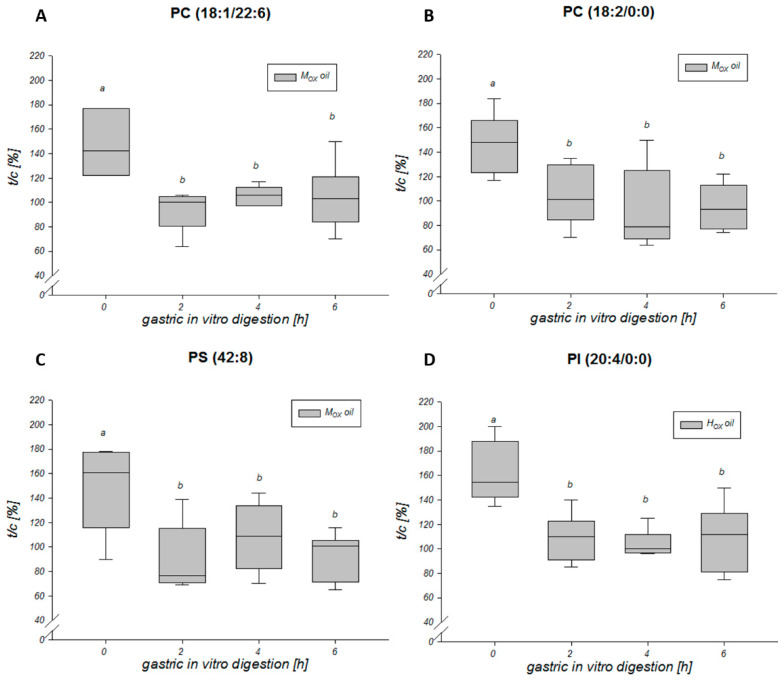
Effect of the oxidized lipids from the grape seed oils digested in vitro for 0, 2, 4, and 6 h on phospholipids in the HGT-1 cells. The phospholipids (**A**) phosphocholines PC (18:1/22:6), (**B**) PC (18:2/0:0) and (**C**) phosphoserine PS (42:8) were modulated by treatment of the HGT-1 cells with the oxidized lipids from the M_OX_ grape seed oil for six hours, whereas (**D**) phosphoinositol PI (20:4/0:0) was upregulated by the incubation of cells with the oxidized lipids from the H_OX_ grape seed oil for six hours. Statistically significant differences (One-Way-ANOVA on ranks, Student–Newmann–Keuls, *p* < 0.05) between the time of digestion (0, 2, 4, 6 h) are indicated by different letters.

A total of four different phospholipids, namely phosphocholines PC (18:1/22:6) and PC (18:2/0:0), phosphoserine PS (42:8) and phosphoinositol PI (20:4/0:0) could be shown to be modulated by the undigested study oils. Data from human studies are lacking and the literature is scarce. However, Bernhard et al. [57] analyzed the composition of the phospholipids in rat and pig mucosa and showed that PC was one of the major phospholipids identified in the gastric mucosa, followed by substantial amounts of PI [57]. Nardone et al. [58], who analyzed the phospholipid composition of gastric mucosa from endoscopic biopsy specimens, showed the same distribution with PC being one of the most abundant phospholipids [58]. In the current study, two PCs and PS were upregulated by about 40%–60% after treatment of the HGT-1 cells with the polar fraction of the undigested M_OX_ oil for six hours, whereas the abundance of the PI was increased by about 50% after the incubation of the gastric cells with the polar fractions of the undigested H_OX_ oil (Figure 4).

The identification of the four phospholipids was performed by high-resolution MS and MS/MS fragmentation. For PC (18:1/22:6) and PC (18:2/0:0), it was the (M + H)^+^ that could be identified in the MS spectrum, whereas for PS (42:8) and PI (20:4/0:0), it was the (M − H)^−^. For the MS/MS measurements, the precursor ion of PC (18:1/22:6) was *m/z* 832.5844. For the fragment *m/z* 814.80, a water molecule was released, at *m/z* 773.66 trimethylamine, at *m/z* 745.65 the choline moiety and at *m/z* 649.63 phosphocholine was cleaved off the molecule. The phosphocholine PC (18:2/0:0) was detected at *m/z* 520.3396. For the fragment *m/z* 502.41, a water molecule was released and at *m/z* 184.10 the phosphocholine molecule could be identified. The phosphoserine PS (42:8) was detected at *m/z* 858.5274. Four different product ions could be identified. At *m/z* 840.76 a water molecule, at *m/z* 771.59 serine, at *m/z* 481.54 the sn1–acyl–chain with serine and at *m/z* 461.38 the sn2–acyl–chain with serine was cleaved off. The precursor ion from the phosphoinositol PI (20:4/0:0) was *m/z* 619.2894. At *m/z* 601.49 a water molecule, at *m/z* 457.31 inositol and at *m/z* 333.21 the sn1–acyl–chain was cleaved off. Another water molecule was cleaved from the glycerophosphoinositol to yield *m/z* 315.09. The inositol–phosphate ion was detected at *m/z* 259.28. At *m/z* 241.06 a water molecule was cleaved from the inositol–phosphate, whereas at *m/z* 223.11 a further water molecule was released.

It could be demonstrated that these four phospholipids were upregulated when the cells were treated with the undigested oxidized lipids (Figure 4), indicating that undigested oxidized lipids affected the composition of the cellular phospholipids by increasing the amount of phospholipids with unsaturated fatty acids. This effect was not seen when the oxidized lipids from the grape seed oil were subjected to in vitro digestion prior to the treatment of gastric cells. Hence, a decrease in the amount of oxTAGs due to the in vitro digestion resulted in a loss of the effect. The effects regarding the composition of phospholipids might not be exclusively assigned to oxTAGs but also to other primary lipid oxidation products. In addition, secondary lipid oxidation and hydrolysis products might be the reason why the effect was abrogated.

The phospholipid-regulating effect might also be provoked by the total peroxides of the M_OX_ oil which increased during in vitro digestion. Three out of four phospholipids were influenced by the oxidized lipids of the M_OX_ oil, of which two of them were PCs, the main class of phospholipids in the gastric cells [57]. However, the peroxide value from the M_OX_ oil decreased after the first two hours of the in vitro digestion and raised only afterwards, thereby excluding an essential role of total peroxides in modulating the cellular lipids. In addition, the peroxide value in the H_OX_ oil decreased after four hours and six hours of the in vitro digestion, which suggests as well that the changes in composition arose from the oxTAGs rather than from other hydroperoxidized lipids. These other oxidized lipids, such as oxidized fatty acids, monoacylglycerols and diacylglycerols, seem to have less or no influence on the cellular phospholipids.

**Figure 5:**
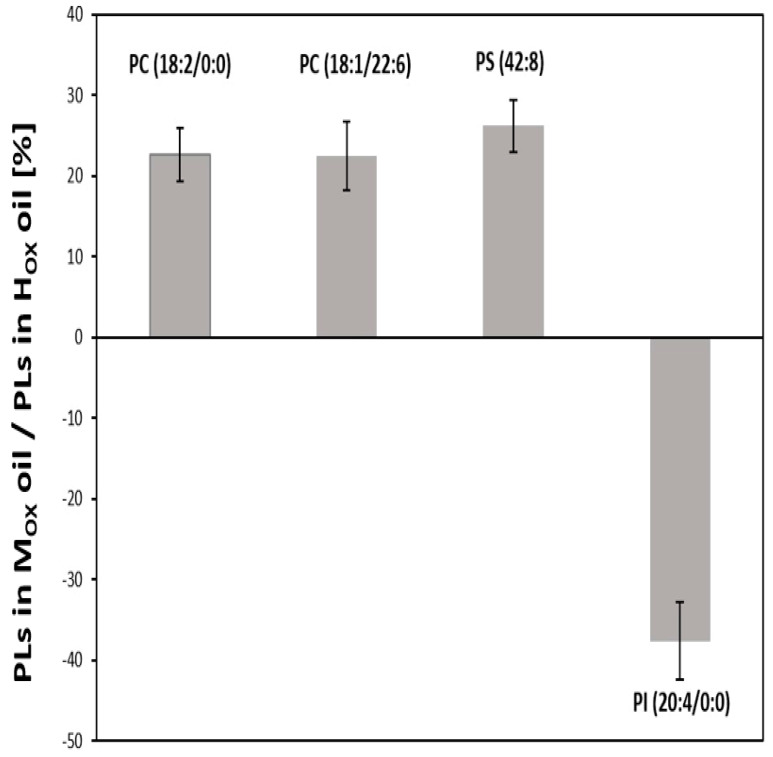
Upregulation of phospholipids (PLs) (%) in the human gastric tumor cells (HGT-1) after incubation with the undigested polar fraction of the M_OX_ grape seed oil in relation to the undigested polar fraction of the H_OX_ grape seed oil (M_OX_/H_OX_).

The oxidized lipids from the M_OX_ and H_OX_ grape seed oils, which differed in their oxidation status, resulted in a different impact on the phospholipids (Figure 5).

The phospholipids were shown to be upregulated by the oxidized lipids in the M_OX_ oil by approximately 20% compared to the oxidized lipids in the H_OX_ oil. The phospholipids could be assigned to the class of phosphocholine and to the class of phosphoserine, as two PCs, PC (18:2/0:0) and PC (18:1/22:6), and one PS, namely PS (42:8), were upregulated. Regarding the phospholipids affected by the polar fraction of the H_OX_ oil, a different result emerged. Contrary to the M_OX_ oil, the class of phosphoinositol, specifically PI (20:4/0:0), was upregulated by about 40% by the undigested polar fraction of the H_OX_ oil in relation to the undigested polar fraction of the M_OX_ oil, thus confirming the different effects resulting from oils with different oxidation status.

## 4. Conclusions

The absorption of dietary fatty acids was facilitated when phospholipids with polyunsaturated fatty acids were increased in enterocytes, but limited absorption was reported when the membranes consisted of mainly saturated fatty acids [7]. In the current study, phospholipids with PUFAs were enhanced by oxTAGs from grape seed oil. After the in vitro digestion of the two different grape seed oils, the phospholipid-regulating effect disappeared, suggesting that oxTAGs might influence phospholipids whereas advanced lipid oxidation products, like aldehydes, which increase during digestion, might not be able to affect the phospholipid composition. Advanced lipid oxidation, like in the H_OX_ oil or after digestion, might inhibit the phospholipid-regulating effect, as the in vitro digested oil did not affect the phospholipid composition. Thus, oxTAGs, similarly to TAGs, might facilitate the passive diffusion of dietary lipids by remodeling phospholipids to increase the proportion of phospholipids with PUFAs, as opposed to secondary lipid oxidation products. Future studies are warranted to elicit the role of oxTAGs in the passive lipid absorption.

## Figures and Tables

**Table 1:** Characterization of the cold-pressed grape seed oils regarding their fatty acid compositions (g/100g oil), peroxide values (meq O_2_/kg oil), hexanal (µg/mL oil) and the amount of tocopherols (mg/kg oil) ^1^.

Fatty Acids	M_OX_ (g/100 g oil)	H_OX_ (g/100 g oil)
C 16:0	6.70 ± 0.33 ^a^	7.39 ± 0.19 ^b^
C 18:0	3.28 ± 0.16 ^a^	3.39 ± 0.09 ^a^
C 18:1	11.1 ± 0.54 ^a^	14.1 ± 0.42 ^b^
C 18:2	78.1 ± 3.86 ^a^	74.0 ± 1.92 ^b^
C 18:3	0.37 ± 0.02 ^a^	0.90 ± 0.02 ^b^
SFA^2^	9.98 ± 0.49 ^a^	10.8 ± 0.28 ^b^
MUFA^3^	11.1 ± 0.54 ^a^	14.1 ± 0.42 ^b^
PUFA	78.4 ± 3.88 ^a^	74.9 ± 1.94 ^b^
total	99.6 ± 4.91 ^a^	99.8 ± 2.63 ^a^
**peroxide value**	**M_OX_ (meq O_2_/kg oil)**	**H_OX_ (meq O_2_/kg oil)**
	18.8 ± 0.39 ^a^	28.2 ± 0.39 ^b^
**hexanal**	**M_OX_ (µg/mL oil)**	**H_OX_ (µg/mL oil)**
	13.6 ± 0.61 ^a^	10.7 ± 0.54 ^b^
**tocopherols**	**M_OX_ (mg/kg oil)**	**H_OX_ (mg/kg oil)**
α-tocopherol	325 ± 43.2 ^a^	329 ± 28.5 ^a^
γ-tocopherol	52.2 ± 3.46 ^a^	80.4 ± 2.12 ^b^
δ-tocopherol	<LOQ	<LOQ
total	410 ± 52.7 ^a^	454 ± 18.8 ^a^

^1^ Statistically significant differences are indicated by different letters within rows. Fatty acid composition is depicted as the mean ± SD (*n* = 4, Student’s *t*-test or Mann–Whitney U-test, *p* < 0.05), peroxide value as the mean ± SD (*n* = 3, Student’s *t*-test, *p* < 0.001) and the amount of tocopherols as the mean ± SD (*n* = 3, Student’s *t*-test, *p* < 0.05). ^2^ saturated fatty acids. ^3^ monounsaturated fatty acids.

**Table 2:** Effects of the gastric in vitro digestion of the cold-pressed grape seed oils on single polyphenols, measured by high-resolution LC-MS (<5 ppm) ^1^ as area under the curve (AUC).

M_OX_-Polyphenols	Catechin (AUC × 10^4^)	Change (%)	Ferulic Acid (AUC × 10^5^)	Change (%)	p-Coumaric Acid (AUC × 10^6^)	Change (%)
(M - H)-	289.0712		193.0501		163.0395	
0 h	18.8 ± 2.23 ^a^		28.3 ± 3.63 ^a^		16.2 ± 2.25 ^a^	
2 h	4.28 ± 0.57 ^b^	−77.3	8.41 ± 1.10 ^b^	−70.3	4.41 ± 0.86 ^b^	−72.8
4 h	4.69 ± 0.63 ^b^	−75.1	9.11 ± 0.19 ^b^	−67.8	4.90 ± 0.54 ^b^	−69.7
6 h	4.48 ± 0.03 ^b^	−76.2	9.71 ± 0.47 ^b^	−65.7	4.76 ± 0.31 ^b^	−70.6
**H_OX_-polyphenols**	**Catechin (AUC × 10^4^)**	**Change (%)**	**Ferulic acid (AUC × 10^5^)**	**Change (%)**	**p-Coumaric acid (AUC × 10^6^)**	**Change (%)**
(M - H)-	289.0712		193.0501		163.0395	
0 h	21.8 ± 2.56 ^a^		45.15 ± 4.66 ^a^		25.0 ± 1.91 ^a^	
2 h	3.25 ± 0.41 ^b^	−85.1	15.3 ± 0.76 ^b^	−66.0	6.91 ± 0.47 ^b^	−72.3
4 h	3.97 ± 0.47 ^b^	−81.8	17.0 ± 2.55 ^b^	−62.4	7.57 ± 0.90 ^b^	−69.7
6 h	3.45 ± 0.27 ^b^	−84.2	15.6 ± 1.23 ^b^	−65.4	7.34 ± 0.66 ^b^	−70.6

^1^ Statistically significant differences are indicated by different letters within rows (*n* = 3, mean ± SD, One-Way-ANOVA, Holm–Sidak, *p* < 0.05).

**Table 3:** Effects of gastric in vitro digestion of cold-pressed grape seed oils on fatty acid composition (g/100 g oil) and the amount of tocopherols (mg/kg oil) ^1^.

M_OX_-Fatty Acids	0 h (g/100 g oil)	6 h (g/100 g oil)	Change (%)	
C 16:0	6.70 ± 0.33 ^a^	6.18 ± 0.16 ^a^	−7.76	
C 18:0	3.29 ± 0.16 ^a^	3.21 ± 0.06 ^a^	−2.43	
C 18:1	11.1 ± 0.54 ^a^	10.7 ± 0.14 ^a^	−4.22	
C 18:2	78.1 ± 3.86 ^a^	72.9 ± 0.80 ^b^	−6.66	
C 18:3	0.36 ± 0.02 ^a^	0.37 ± 0.01 ^a^	+2.77	
SFA	9.98 ± 0.49 ^a^	10.0 ± 0.14 ^a^	+0.30	
MUFA	11.1 ± 0.54 ^a^	10.7 ± 0.14 ^a^	−4.22	
PUFA	78.4 ± 3.88 ^a^	73.2 ± 0.80 ^b^	−6.63	
total	99.6 ± 4.91 ^a^	93.9 ± 1.07 ^b^	−5.66	
**M_OX_-Tocopherols**	**0 h (mg/kg oil)**	**2 h (mg/kg oil)**	**4 h (mg/kg oil)**	**6 h (mg/kg oil)**
α-tocopherol	325 ± 43.2 ^a^	314.3 ± 6.88 ^a^	321 ± 7.74 ^a^	318 ± 7.74 ^a^
γ-tocopherol	52.2 ± 3.46 ^a^	51.8 ± 3.77 ^a^	53.6 ± 2.44 ^a^	52.4 ± 1.39 ^a^
δ-tocopherol	<LOQ	<LOQ	<LOQ	<LOQ
total	410 ± 52.7 ^a^	416 ± 16.3 ^a^	417 ± 7.75 ^a^	400 ± 17.7 ^a^
**H_OX_-Fatty Acids**	**0 h (g/100 g oil)**	**6 h (g/100 g oil)**	**Change (%)**	
C 16:0	7.39 ± 0.19 ^a^	7.42 ± 0.07 ^a^	+0.41	
C 18:0	3.39 ± 0.09 ^a^	3.27 ± 0.03 ^a^	−3.54	
C 18:1	14.1 ± 0.42 ^a^	13.3 ± 0.11 ^b^	−6.02	
C 18:2	74.0 ± 1.92 ^a^	68.6 ± 0.56 ^b^	−7.32	
C 18:3	0.90 ± 0.02 ^a^	0.78 ± 0.01 ^b^	−13.3	
SFA	10.8 ± 0.28 ^a^	10.7 ± 0.10 ^a^	−0.93	
MUFA	14.1 ± 0.42 ^a^	13.3 ± 0.11 ^b^	−5.67	
PUFA	74.9 ± 1.94 ^a^	69.4 ± 0.56 ^b^	−7.34	
total	99.8 ± 2.63 ^a^	93.3 ± 0.75 ^b^	−6.49	
**H_OX_-Tocopherols**	**0 h (mg/kg oil)**	**2 h (mg/kg oil)**	**4 h (mg/kg oil)**	**6 h (mg/kg oil)**
α-tocopherol	329 ± 28.5 ^a^	330 ± 9.27 ^a^	330 ± 3.64 ^a^	338 ± 8.25 ^a^
γ-tocopherol	80.4 ± 2.12 ^a^	86.3 ± 3.92 ^a^	84.1 ± 5.82 ^a^	87.3 ± 7.43 ^a^
δ-tocopherol	< LOQ	< LOQ	93.6 ± 9.18 ^a^	88.6 ± 12.9 ^a^
total	454 ± 18.8 ^a^	471 ± 15.3 ^a^	510 ± 12.2 ^b^	514 ± 10.9 ^b^

^1^ Statistically significant differences are indicated by different letters within rows. Fatty acid composition is depicted as the mean ± SD (*n* = 4, Student’s *t*-test or Mann–Whitney U-test, *p* < 0.05) and the amount of tocopherols as the mean ± SD (*n* = 3, One-Way-ANOVA, Holm–Sidak, *p* < 0.05).

## Supplementary Materials

The Supplementary Materials are available online at https://www.mdpi.com/2218-273X/10/5/708/s1. Table S1: Analysis of oxidized TAGs in grape seed oil by LC-MS, Table S2: Analysis of free linoleic acid, linoleic acid hydroxide and linoleic acid hydroperoxide by LC-MS, Table S3: Analysis of phytosterols, their esters with linoleic acid and phospholipids in grape seed oil by high-resolution LC-MS.

## References

[1] Shahidi F., Zhong Y. (2010). Lipid oxidation and improving the oxidative stability. Chem. Soc. Rev..

[2] Abbott S.K., Else P.L., Hulbert A.J. (2010). Membrane fatty acid composition of rat skeletal muscle is most responsive to the balance of dietary n-3 and n-6 PUFA. Br. J. Nutr..

[3] Lutterodt H., Slavin M., Whent M., Turner E., Yu L. (2011). Fatty acid composition, oxidative stability, antioxidant and antiproliferative properties of selected cold-pressed grape seed oils and flours. Food Chem..

[4] Choe E., Min D.B. (2006). Mechanisms and Factors for Edible Oil Oxidation. Compr. Rev. Food Sci. Food Saf..

[5] Kanner J. (2007). Dietary advanced lipid oxidation endproducts are risk factors to human health. Mol. Nutr. Food Res..

[6] Frey L., Hiller S., Riek R., Bibow S. (2018). Lipid- and Cholesterol-Mediated Time-Scale-Specific Modulation of the Outer Membrane Protein X Dynamics in Lipid Bilayers. J. Am. Chem. Soc..

[7] Wang B., Rong X., Duerr M.A., Hermanson D.J., Hedde P.N., Wong J.S., Vallim T.Q., Cravatt B.F., Gratton E., Ford D.A. (2016). Intestinal Phospholipid Remodeling Is Required for Dietary-Lipid Uptake and Survival on a High-Fat Diet. Cell Metab..

[8] Zaunschirm M., Pignitter M., Kopic A., Keßler C., Hochkogler C., Kretschy N., Somoza M.M., Somoza V. (2019). Exposure of Human Gastric Cells to Oxidized Lipids Stimulates Pathways of Amino Acid Biosynthesis on a Genomic and Metabolomic Level. Molecules.

[9] Koziolek M., Carriere F., Porter C.J.H. (2018). Lipids in the Stomach—Implications for the Evaluation of Food Effects on Oral Drug Absorption. Pharm. Res..

[10] Kanner J., Lapidot T. (2001). The stomache as a bioreactor: Dietary lipid peroxidation in the gastric fluid and the effects of plant-derived antioxidants. Free Radical. Bio. Med..

[11] Ohnishi M., Hirose S., Kawaguchi M., Ito S., Fujino Y. (2014). Chemical Composition of Lipids, Especially Triacylglycerol, in Grape Seeds. Agric. Biol. Chem..

[12] Demirtas I., Pelvan E., Özdemir İ.S., Alasalvar C., Ertas E. (2013). Lipid characteristics and phenolics of native grape seed oils grown in Turkey. Eur. J. Lipid. Sci. Tech..

[13] Pignitter M., Dumhart B., Gartner S., Jirsa F., Steiger G., Kraemer K., Somoza V. (2014). Vitamin A is rapidly degraded in retinyl palmitate-fortified soybean oil stored under household conditions. J. Agric. Food. Chem..

[14] Grüneis V., Fruehwirth S., Zehl M., Ortner J., Schamann A., König J., Pignitter M. (2019). Simultaneous Analysis of Epoxidized and Hydroperoxidized Triacylglycerols in Canola Oil and Margarine by LC-MS. J. Agric. Food Chem..

[15] Wheeler D.H. (1932). Peroxide formation as a measure of autoxidative deterioriation. Oil Soap.

[16] Versantvoort C.H., Oomen A.G., Van de Kamp E., Rompelberg C.J., Sips A.J. (2005). Applicability of an in vitro digestion model in assessing the bioaccessibility of mycotoxins from food. Food Chem. Toxicol..

[17] Nieva-Echevarría B., Goicoechea E., Manzanos M.J., Guillen M.D. (2016). A study by (1)H NMR on the influence of some factors affecting lipid in vitro digestion. Food Chem..

[18] Cortot A., Phillips S.F., Malagelada J.-R. (1981). Gastric emptying of lipids after ingestion of a solid-liquid meal in humans. Gastroenterology.

[19] Márquez-Ruiz G., Jorge N., Martín-Polvillo M., Dobarganes M.C. (1996). Rapid, quantitative determination of polar compounds in fats and oils by solid-phase extraction and size-exclusion chromatography using monostearin as internal standard. J. Chromatogr. A.

[20] ICH (1996). International Conference on Harmonization of Technical Requirements for the Registration of Pharmaceuticals for Human Use, Validation of Analytical Procedures: Text and Methodology Q2(R1).

[21] Shrivastava A., Gupta V.B. (2011). Methods for the determination of limit of detection and limit of quantitation of the analytical methods. Chron. Young Sci..

[22] Giuffrida F., Golay P.-A., Destaillats F., Hug B., Dionisi F. (2005). Accurate determination of hexanal in beef bouillons by headspace solid-phase microextraction gas-chromatography mass-spectrometry. Eur. J. Lipid. Sci. Tech..

[23] Pignitter M., Stolze K., Gartner S., Dumhart B., Stoll C., Steiger G., Kraemer K., Somoza V. (2014). Cold fluorescent light as major inducer of lipid oxidation in soybean oil stored at household conditions for eight weeks. J. Agric. Food Chem..

[24] Singleton V.L., Rossi J.A. (1965). Colorimetry of total phenolics with phosphomolybdic-phosphotungstic and reagents. Am. J. Enol. Viticult..

[25] Liszt K.I., Ley J.P., Lieder B., Behrens M., Stoger V., Reiner A., Hochkogler C.M., Kock E., Marchiori A., Hans J. (2017). Caffeine induces gastric acid secretion via bitter taste signaling in gastric parietal cells. Proc. Natl. Acad. Sci. USA.

[26] Stoeger V., Liszt K.I., Lieder B., Wendelin M., Zopun M., Hans J., Ley J.P., Krammer G.E., Somoza V. (2018). Identification of Bitter-Taste Intensity and Molecular Weight as Amino Acid Determinants for the Stimulating Mechanisms of Gastric Acid Secretion in Human Parietal Cells in Culture. J. Agric. Food Chem..

[27] Liszt K.I., Eder R., Wendelin S., Somoza V. (2015). Identification of Catechin, Syringic Acid, and Procyanidin B2 in Wine as Stimulants of Gastric Acid Secretion. J. Agric. Food Chem..

[28] Hoi J.K., Lieder B., Pignitter M., Hans J., Ley J.P., Lietard J., Hoelz K., Somoza M., Somoza V. (2019). Identification of Cinnamaldehyde as Most Effective Fatty Acid Uptake Reducing Cinnamon-Derived Compound in Differentiated Caco-2 Cells Compared to Its Structural Analogues Cinnamyl Alcohol, Cinnamic Acid, and Cinnamyl Isobutyrate. J. Agric. Food Chem..

[29] Zhang H., Gao Y., Sun J., Fan S., Yao X., Ran X., Zheng C., Huang M., Bi H. (2017). Optimization of lipid extraction and analytical protocols for UHPLC-ESI-HRMS-based lipidomic analysis of adherent mammalian cancer cells. Anal. Bioanal. Chem..

[30] Pignitter M., Zaunschirm M., Lach J., Unterberger L., Kopic A., Kessler C., Kienesberger J., Pischetsrieder M., Eggersdorfer M., Riegger C. (2018). Regioisomeric distribution of 9- and 13-hydroperoxy linoleic acid in vegetable oils during storage and heating. J. Sci. Food Agric..

[31] Ovcharova T., Zlatanov M., Ivanov A. (2014). Changes in grape seed oil during fermentation. Eur. Int. J. Sci. Technol..

[32] Tautenhahn R., Patti G.J., Rinehart D., Siuzdak G. (2012). XCMS Online: A Web-Based Platform to Process Untargeted Metabolomic Data. Anal. Chem..

[33] Chambers M.C.M.B., Burke R., Amodei D., Ruderman D.L., Neumann S., Gatto L., Fischer B., Pratt B., Egertson J., Hoff K. (2012). A cross-platform toolkit for mass spectrometry and proteomics. Nat. Biotechnol..

[34] Prince J.T., Marcotte E.M. (2006). Chromatographic Alignment of ESI-LC-MS Proteomics Data Sets by Ordered Bijective Interpolated Warping. Anal. Chem..

[35] Tautenhahn R., Bottcher C., Neumann S. (2008). Highly sensitive feature detection for high resolution LC/MS. BMC Bioinform..

[36] Beveridge T.H.J., Girard B., Kopp T., Drover J.C.G. (2005). Yield and composition of grape seed oils extracted by supercritical carbon dioxide and petroleum ether: Varietal effects. J. Agric. Food Chem..

[37] Sabir A., Unver A., Kara Z. (2012). The fatty acid and tocopherol constituents of the seed oil extracted from 21 grape varieties (Vitis spp.). J. Agric. Food Chem..

[38] Maszewska M., Florowska A., Dluzewska E., Wroniak M., Marciniak-Lukasiak K., Zbikowska A. (2018). Oxidative Stability of Selected Edible Oils. Molecules.

[39] The-Codex-Alimentarius (2001). Joint FAO/WHO Food Standards Programme Codex Alimentarius Commission.

[40] Shahidi F., Pegg R.B. (1994). Hexanal as an Indicator of the Flavor Deterioration of Meat and Meat Products. Lipids in Food Flavors.

[41] Fernandes L., Casal S., Cruz R., Pereira J.A., Ramalhosa E. (2013). Seed oils of ten traditional Portuguese grape varieties with interesting chemical and antioxidant properties. Food Res. Int..

[42] Assumpção C.F., Nunes I.L., Mendonça T.A., Bortolin R.C., Jablonski A., Flôres S.H., de Oliveira Rios A. (2015). Bioactive Compounds and Stability of Organic and Conventional Vitis labrusca Grape Seed Oils. J. Am. Oil Chem. Soc..

[43] Vieira S.A., Zhang G., Decker E.A. (2017). Biological Implications of Lipid Oxidation Products. J. Am. Chem. Soc..

[44] Kenmogne-Domguia H.B., Moisan S., Viau M., Genot C., Meynier A. (2014). The initial characteristics of marine oil emulsions and the composition of the media inflect lipid oxidation during in vitro gastrointestinal digestion. Food Chem..

[45] Chew S.-C., Tan C.-P., Long K., Nyam K.-L. (2015). In-vitro evaluation of kenaf seed oil in chitosan coated-high methoxyl pectin-alginate microcapsules. Ind. Crops Prod..

[46] Cheong A.M., Tan C.P., Nyam K.L. (2016). In-vitro gastrointestinal digestion of kenaf seed oil-in-water nanoemulsions. Ind. Crops Prod..

[47] Garavaglia J., Markoski M.M., Oliveira A., Marcadenti A. (2016). Grape Seed Oil Compounds: Biological and Chemical Actions for Health. Nutr. Metab. Insight.

[48] Bjelica M., Vujasinović V., Rabrenović B., Dimić S. (2019). Some Chemical Characteristics and Oxidative Stability of Cold Pressed Grape Seed Oils Obtained from Different Winery Waste. Eur. J. Lipid. Sci. Tech..

[49] Choe E., Min D.B. (2009). Mechanisms of Antioxidants in the Oxidation of Foods. Compr. Rev. Food Sci. Food Saf..

[50] Dai F., Chen W.F., Zhou B. (2008). Antioxidant synergism of green tea polyphenols with alpha-tocopherol and L-ascorbic acid in SDS micelles. Biochimie.

[51] Réblová Z., Okrouhlá P. (2010). Ability of Phenolic Acids to Protect α-Tocopherol. Czech J. Food Sci..

[52] Pignitter M., Somoza V. (2012). Critical evaluation of methods for the measurement of oxidative rancidity in vegetable oils. J. Food Drug Anal..

[53] Barriuso B., Astiasarán I., Ansorena D. (2012). A review of analytical methods measuring lipid oxidation status in foods: A challenging task. Eur. Food Res. Technol..

[54] N’Goma J.-C.B., Amara S., Dridi K., Jannin V., Carrière F. (2012). Understanding the lipid-digestion processes in the GI tract before designing lipid-based drug-delivery systems. Ther. Deliv..

[55] Armand M. (2007). Lipases and lipolysis in the human digestive tract: Where do we stand?. Curr. Opin. Clin. Nutr. Metab. Care.

[56] Elisia I., Kitts D.D. (2011). Quantification of hexanal as an index of lipid oxidation in human milk and association with antioxidant components. J. Clin. Biochem. Nutr..

[57] Bernhard W., Postle A.D., Linck M., Sewing K.-F. (1994). Composition of phospholipid classes and phosphatidylcholine molecular species of gastric mucosa and mucus. Biochim. Biophys. Acta.

[58] Nardone G., Laccetti P., Civiletti C., Budillon G. (1993). Phospholipid composition of human gastric mucosa: A study of endoscopic biopsy specimens. Gut.

