# Supplementary material for: In Vitro Digestion of Grape Seed Oil Inhibits Phospholipid-Regulating Effects of Oxidized Lipids
Source: Biomolecules. 2020 May 2;10(5):708. doi: 10.3390/biom10050708 (PMC7277833; doi:10.3390/biom10050708)
Supplement: Supplementary file 1 [file biomolecules-10-00708-s001.pdf]

SUPPORTING INFORMATION

Sarah Fruehwirth [1], Sofie Zehentner [1], Mohammed Salim [1], Sonja Sterneder [1], Johanna Tiroch [1], Barbara Lieder [1], Martin Zehl [2], Veronika Somoza [1] and Marc Pignitter [1]*

[1] Department of Physiological Chemistry, Faculty of Chemistry, University of Vienna, 1090 Vienna, Austria.
[2] Department of Analytical Chemistry, Faculty of Chemistry, University of Vienna, 1090 Vienna, Austria; martin.zehl@univie.ac.at.
* Correspondence: marc.pignitter@univie.ac.at; +43 14277 70621

Table S1. Analysis of oxidized TAGs in grape seed oil by LC-MS

[M+NH_4]$^+$ adducts	oxTAGs precursor ion m/z	product ion m/z
**TAG 54:3 [OOH]**	934.8	635.5<br>617.5<br>603.5<br>601.5
**TAG 54:4 [OOH]**	932.8	633.5<br>615.5<br>603.5<br>601.5
**TAG 54:5 [OOH]**	930.7	633.5<br>631.5<br>615.5<br>613.5<br>603.5<br>601.5
**TAG 54:6 [OOH]**	928.7	631.5<br>629.5<br>617.5<br>613.5<br>611.5<br>601.5<br>599.5
**TAG 54:0 [O]**	922.8	621.5<br>605.5<br>603.5
**TAG 54:1 [O]**	920.8	621.5<br>619.5<br>617.5<br>603.5<br>601.5<br>339.3
**TAG 54:2 [O]**	918.8	619.5<br>617.5<br>603.5<br>601.5
**TAG 54:3 [O]**	916.7	617.5<br>603.5<br>601.5<br>599.5
**TAG 54:4 [O]**	914.8	617.5<br>615.5<br>601.5<br>599.5<br>597.5

Table S2. Analysis of free linoleic acid, linoleic acid hydroxide and linoleic acid hydroperoxide by LC-MS.

$[M-H]^-$ ions	precursor ion m/z	product ion m/z
free linoleic acid	279.2	261.2
		59.0
		71.0
linoleic acid hydroxide	295.5	277.3
		195.5
linoleic acid hydroperoxide	311.0	113.0

Table S3. Analysis of phytosterols, their esters with linoleic acid and phospholipids in grape seed oil by high-resolution LC-MS

$[M+H]^+$ ions	phytosterols precursor ion m/z	esters with linoleic acid precursor ion m/z
sitosterol	415.3940	677.6236
campesterol	401.3783	663.6079
stigmasterol / Δ5-avenasterol	413.3783	675.6079
cholesterol	387.3627	649.5923
cholestanol	389.3783	651.6079
brassicasterol	399.3627	661.5923
campestanol	403.349	665.6236
sitostanol	417.4096	679.6392
**phospholipids**	**precursor ion m/z**	
PI(18:2/18:1)	860.5415	
PI(18:2/18:2)	858.5258	
PI(16:0/18:1)	836.5415	
PI(16:0/16:0)	810.5258	
PI(16:0/18:2)	834.5258	
PC(18:1/18:1)	785.5935	
PC(14:0/14:0)	677.4996	
PC(14:0/18:1)	731.5465	
